# Primary health care reforms in Slovenia: leveraging existing structures to expand care: Pia Vracko

**DOI:** 10.1093/eurpub/ckac129.627

**Published:** 2022-10-25

**Authors:** K Polin, G Scarpetti, T Albreht, V Petrič, P Vracko

**Affiliations:** 1 Department of Health Care Management, Technical University Berlin, Berlin, Germany; 2 European Observatory on Health Systems and Policies, Technical University Berlin, Berlin, Germany; 3 National Institute of Public Health, Ljubljana, Slovenia; 4 Health Promotion and Prevention, Ministry of Health, Ljubljana, Slovenia

## Abstract

Primary health care (PHC) in Slovenia is delivered mainly by a network of 63 public community-based primary health care centres (CPHCs), serving as entry points to the health system. Here, multidisciplinary teams provide an array of preventative, diagnostic, therapeutic, palliative, and health promotion services under one roof. Since 2011, several reforms in PHC highlight integrated care. A national scale-up of Family Medicine Practices is underway, where all family medicine teams include a 0.5 FTE registered nurse to improve prevention, early diagnosis and care coordination of chronic patients. Health promotion centers (HPCs) are being introduced in CPHCs to support people in healthy lifestyle, with currently 28 HPCs managed by CPHCs and supported operationally by the National Institute of Public Health. New mental health centers facilitate access to comprehensive mental health care. In 2020, dedicated temporary COVID-19 units in CPHCs played a key role in treating mild/moderate cases and shielding hospitals from overburden. Regarding implementation, pilots have been critical to creating a strong evidence base to enable sustainable (sometimes external) financing, while innovations capitalize on existing links between Slovenia's primary care and public health functions and the Ministry of Health for governance and the flexibility of the multidisciplinary, multiple-practice care model represented by CPHCs. Though this has eased their initial introduction into existing structures, challenges remain. These include dissatisfaction among family physicians due to high administrative burden and an outdated CPHC governance model that limits managers’ authority as well as workforce shortages in public health and primary care. Financial incentives, task shifting, and adjustments to education and training have been used to mitigate these issues. Slovenia's experience may serve as a case study for countries interested in improving their primary healthcare services.

